# Medical genetics teaching in Iranian medical schools, especially Ahvaz, south of Iran

**Published:** 2014-04

**Authors:** MAHDI BIJANZADEH

**Affiliations:** Department of Medical Genetics, School of Medicine, Ahvaz Jundishapur University of Medical Sciences, Ahvaz, Iran

**Keywords:** Medical students, Education, Genetics, Questionnaires

## Abstract

**Introduction**: Physicians have to visit, diagnose and refer patients with genetic disorders, so they need to be familiar with the basics and indications of genetic tests. In other words, they should have effective theoretical and practical knowledge about medical genetics before they do their job. Medical genetics courses at Medical Universities of Iran are generally presented as a theoretical subject in the first period of medical education.

**Methods**: In this descriptive research, the results of interviews with teachers of medical genetics in 30 medical schools in Islamic Republic of Iran and responses to a questionnaire by 125 medical students of Ahvaz Jundishapour University of medical sciences, about presentation time, curricula and also efficacy of medical genetics courses were analyzed. The interviews with teachers were done on phone and the students’ comments were collected by a researcher-made questionnaire. The data were analyzed, using SPSS software, version 14.

**Results**: In two thirds of medical universities, medical genetics is taught in the third or fourth semester and in 5 universities in the fifth semester. 86% of the students believed that the quality of genetics courses is moderate and such courses are very beneficial to medical students.

**Conclusion**: This article suggests that medical genetics be offered in the second or third period of medical education (physiopathology or stagger period). Furthermore, in teaching such courses advanced educational methods (animation presentation, case-based learning, problem-based learning, etc.) should be used, together with simple genetic tests in laboratories, and the visit of genetic patients in hospitals and genetics centers.

## Introduction

It is widely accepted that human genetics is central to the bodies of knowledge that are the responsibilities of modern medical schools. However, human genetics educators have become increasingly concerned about the adequacy of human genetics components of medical school curricula (1, 2). One step in dealing with this concern is to determine whether there is a real discrepancy between what is taught and what could and should be taught (3). Over the next decade, during the fast growth of new medical information and knowledge, new models of clinical education are required to address the changes in the patient population, disease profile and management strategies (4). In most medical schools of Iran, medical undergraduate education takes a minimum of 7 years traditionally and includes "basic sciences" period, "physiopathology" period (theoretical aspects of different common diseases) "extern or stager" period (learning practical aspects of diseases at the patient’s bed), and "internship" period in which students are responsible for diagnosis, treatment and management of their patients in the hospitals. At the end of the first and third periods, medical students should pass national exams to enter the next periods. In most universities, medical genetics education is restricted to two theoretical units (34 hours) in basic sciences period, during the first 5 semester of medical course (5). Topics of medical genetics curriculum include: Introduction to medical genetics, Nucleic acids and proteins, DNA packaging, Cell division, Mutations, Single gene diseases, Molecular and cellular genetic techniques of diagnosis, Multifactorial diseases, Genetics of cancer, Genetic counseling, Genetic engineering, Gene therapy and Stem cell genetics. It is apparent that there exist basic and clinical materials. The basic materials include the theoretical and non-visible basic foundation of medical genetics that should be imagined by students. Teachers must use educational aids such as pictures, movies, animations and models for better understanding of basic information. The clinical part includes: 1. Processes of molecular and cellular genetic diagnosis methods that are better to be taught practice-based in laboratories; 2. Genetic disorders including their etiology, manifestations, diagnosis, prognosis and treatment; and 3. Genetics counseling. Therefore, medical genetics is similar to some subjects of basic sciences period such as microbiology, virology and immunology that introduce a lot of clinical disorders whose clinical aspects will be taught in hospitals, and different from other subjects introducing only basic physiology of the body, such as anatomy, physiology, biochemistry and histology. On the other hand, it is similar to some subjects of clinical period of medical study such as internist, surgery, pediatrics, gynecology and infectious diseases (6-9).

The medical school of Ahvaz Jundishapur University of Medical Sciences is an important medical school in south of Iran that ranked as one of the first class medical schools by the Ministry Health, Treatment and Medical Education.

In this article, at first, time and contents of medical genetics teaching in medical schools in Iran will be analyzed through interviews with the teachers. Then the questionnaire answered by medical students about efficacy of the medical genetics course will be analyzed, and finally several methods for better education of this important basic course will be suggested.

## Methods

Interviewing the teaching staff and evaluating the websites of 30 medical universities about the semester in which the genetics course is offered, the content and syllabus of the course in those universities, and the practical items which are taught, we collected the required information about the topic under investigation. This information is displayed in Table 1.

All the students of Ahvaz Jundishapour University of Medical Sciences who had passed medical genetics (first semester of 2012 and first semester of 2013) were selected. These 125 medical students had attended genetics classes based on a common curriculum in the third semester of basic sciences period. At the last session of their medical genetics class, they were asked to answer a valid and reliable questionnaire. The participants were asked about the efficacy of the topics, effects of this course on their clinical ability in diagnosis and referral of genetic disorders and the best time for the course to be offered. Answers to this questionnaire are shown in Table 2. These students received the questionnaire in the last session of their genetics class and all of the students who were present filled the questionnaire and submitted it to the researcher in the same session (response rate was 100%).

The purpose of this study was to investigate the views of medical students of Ahvaz Jundishapour University of medical sciences about time and methods of medical genetics teaching in Iranian medical schools. 

The validity of the questionnair was verified by some expert teachers in education and research, and its reliability was calculated: Cronbach's alpha=0.659. The frequency of the answers was obtained by direct counting. SPSS 14 (SPSS Inc, Chicago, IL, USA) was used for statistical analysis of frequencies and percentages.

The students were not obliged to answer the questionnaire or write their names, so there were no ethical conflicts. 

## Results

Table 1 presents the main semester of basic sciences that medical genetics course is offered in different schools of medical universities in Iran. Around one third of medical universities offer medical genetics in the third semester, one third (10) in the forth semester and 5 universities in the fifth semester of basic sciences. Shahroud and Sabzevar Universities of Medical Sciences offer medical genetics in the first and Hamedan University in the second semester of basic sciences. Shiraz University of Medical Science presents this course in two semesters, the first and third semesters. In Shahid Beheshti University of Medical Sciences although main medical genetics topics are offered in the third semester of basic sciences, students pass 4 practice units (around 100 hours) in pediatrics, gynecology and neurology wards of hospitals and visit patients admitted for genetic disorders. They can also pass one optional practice unit (around 34 hours) in genetic laboratory and learn common methods in cytogenetic and molecular genetics. Tehran, Mashhad, Ahvaz, Kerman, Rasht, Qom, Bojnord, Birjand, Rafsanjan and Ghazvin Universities of Medical Sciences offer medical genetics in the third semester. In Mashhad University of Medical Sciences, where the course is offered in third semester of basic sciences, students can pass an optional practice period in hospitals about genetic disorders. This course is offerd in Isfahan, Tabriz, Sari, Arak, Baboul, Kashan, Ardabil, Zanjan and Shahrekord Universities of Medical Sciences in the fourth and in Yazd, Kermanshah, Ilam, Khorramabad and Gonabad Universities of Medical Sciences in the fifth semester of medical sciences.

**Table 1:** Presentation of medical genetics in Iranian medical schools

**School(s) of medicine**	**Other presentation**	**Time of main presentation**
Shahroud, Sabzevar	-	First semester of basic sciences
Hamedan	-	Second semester of basic sciences
Shiraz	-	First and third semester of basic sciences
Shahid Beheshti	4 units in pediatrics, gynecology & neurology wards 1 unit laboratory (optional)	Third semester of basic sciences
Tehran, Ahvaz, Kerman, Rasht, Qom, Bojnord, Birjand, Rafsanjan, Ghazvin	-	Third semester of basic sciences
Mashhad	In hospital wards (optional)	Forth semester of basic sciences
Isfahan, Tabriz, Sari, Arak, Baboul, Kashan, Ardabil, Zanjan, Shahrekord,	-	Forth semester of basic sciences
Yazd, Kermanshah, Ilam, Khorramabad, Gonabad	Other presentation	Time of main presentation

Table 2 presents the answers of 125 students of Ahvaz Joundishapour University of Medical Sciences to the questionnaire about medical genetics, the course they passed in the third semester. In the first question, the students were asked “how much can medical genetics topics increase your ability in the primary diagnosis of genetic disorders?” 15.2% believed much, and others 84.8% believed nothing, little and to some extent. The second question was “By studying this course how much do you learn about clinical signs and symptoms of genetic disorders?” 43.2% believed little, 48.8% believed to some extent and 8% believed a lot. The third question asked “How much teaching is clinical aspects and manifestation of genetics necessary for medical students?”, 11.2% answered little, 48% answered to some extent and 38.6% answered a lot. The fourth question asked “How much are the current subjects of medical genetics enough for future medical practice?”, 16% believed absolutely not enough, 50.4% believed partially enough, 21.6% believed enough and 12% believed quite enough. The last question asked “Which period of medical study is better for teaching medical genetics?” 14.4% suggested basic sciences (first period), 20% suggested second period (physiopathology), 20% suggested third period and 45.6% believed that it should be taught during two of the above periods.

**Table 2:** Medical students’ responses to the questionnaire about medical genetics teaching

**Question**	**Very less No. (%)**	**Less** **No. (%)**	**Medium** **No. (%)**	**More** **No. (%)**	**Total** **No. (%)**
**Ability of primary diagnosis**	11 (8.8)	40 (32)	55 (44)	19 (15.2)	125 (100)
**Familiarity with clinical manifestation**	15 (12)	39 (31.2)	61 (48.8)	10 (8)	125 (100)
**Necessary for medical students**	1 (0.8)	13 (10.4)	61 (48.8)	50 (40)	125 (100)
**Enough for future job**	**No**	**Partially**	**Yes**	**More**	**Total**
	20 (16)	63 (50.4)	27 (21.6)	15 (12)	125 (100)
**Which study period**	**First**	**Second**	**Third**	**2 of them**	**Total**
	18 (14.4)	25 (20)	25 (20)	57 (45.6)	125 (100)

## Discussion

According to the results of this study, generally all medical schools in Iran offer the medical genetics course in different semesters of the basic sciences period of medical education (Table 1). In this period students do not have enough information about clinical practice, because they generally learn theoretical subjects in medical basic sciences and have non-clinical laboratory classes. This finding is similar to the finding of Virginia and her team after evaluation of the surveys of 149 U.S. and Canadian medical genetics course in 2004. They concluded that most respondents (77%) reported that medical genetics was offered in the first year of medical school; only half (47%) reported that medical genetics was incorporated into the third and fourth years. About two thirds of the respondents (62%) reported that 20 to 40 hours was devoted to medical genetics instruction, which was largely concerned with general concepts (86%) rather than practical application (11%). Topics most commonly taught were: cancer genetics (94.2%), multifactorial inheritance (91.3%), Mendelian disorders (90.3%), clinical cytogenetics (89.3%), and patterns of inheritance (87.4%) (6).

Medical genetics includes theoretical, laboratory and clinical subjects. Clinical subjects are concerned with genetic diseases and should be taught carefully and genetics patients should be visited in hospitals. The students have theoretical classes in basic, laboratory and clinical aspects of medical genetics in the basic sciences period and in the next period of their medical education they do not have any experience in genetics, unless they visit a case incidentally. This is evident in the responses by medical students of Ahvaz Joundishapour University of Medical Sciences (Table 2). The students were not satisfied with their ability for primary diagnosis of genetic disorders (32%, little and 44%, to some extent). 50.4% of them believed that the contents and this type of genetics education were partially enough for future practice of medical students. All of these results showed that current teaching of medical genetics did not improve clinical view and ability of medical students about genetic disorders. Regarding the time the course is offered, 14.4% of the students agreed with the first period of medical education, 20% agreed with the second, 20% with the third and 45.6% believed that medical genetics should be taught during two periods. This implies that medical students do not gain and maintain enough information, ability and skills for the diagnosis and management of medical diseases.

The first suggestion of this article is to offer the medical genetics course in periods that students learn clinical subjects or enter hospitals, i.e. the second or third period of medical education, physiopathology or stagger period (7). If it is not possible, as the second suggestion, during presentation of basic and theoretical topics in the first period, the students should attend laboratory classes to observe molecular and cellular genetic tests, visit genetic patients admitted to the hospital wards and attend genetic counseling meeting held by their teachers.

Because medical genetics has a lot of imaging subjects, and especially in the current situation that the genetics course is presented theoretically and through lecture and in the first period of medical education, this important base of "genomic medicine" should be taught through advanced teaching methods (the third suggestion of this article) (8). These methods are: using pictures, animations, movies, models, ambulatory teaching, problem-based learning, apprenticeship model, small group discussion, large group discussion, computer-assisted learning, role play, role model, video presentation, workshop, task-based teaching and demonstration (9-11). The author experienced a useful topic for presentation: "clinical manifestation of genetic disorders" before teaching genetic disorders and their clinical signs and symptoms. Medical genetics is a practical subject of medicine and similar to internist, surgery, pediatrics, gynecology and other subjects, its special clinical manifestations should be learnt before dealing with details of diseases. In this course special presentations of genetic disorders in each system of human body, for example, head and neck, chest, abdomen, urinogenitalia, extremities, spine and skin, must be defined and explained by photos and clinical evidence (12).

The strength of the current research is the contribution of all medical students who had passed medical genetics course in two successive years in the medical school and its weakness is its limitation to only one medical school, although general information of other main medical schools of the country has been taken.

## Conclusion

Regarding the increasing effects of genetics knowledge in medicine and delivery of "Genomic medicine", medical schools should employ the best methods to provide medical students with sufficient genetics information and skills. This course should be offered when students are gaining clinical experience, i.e. the physiopathology period. Furthermore, advanced teaching methods with counseling sessions and visiting genetics patients admitted to hospitals can be very helpful.

It is useful to evaluate details of medical genetics curriculum in each medical school of medical universities. The materials covered, number of contact hours, what department sponsors the course, mind-remaining and efficacy of each topic on the practice of graduated students are other subjects to investigate in future studies.
